# The differential effects of isoflurane and sevoflurane on neonatal mice

**DOI:** 10.1038/s41598-020-76147-6

**Published:** 2020-11-09

**Authors:** Shuai Zhao, Ziqi Fan, Jing Hu, Yueli Zhu, Caixiu Lin, Ting Shen, Zheyu Li, Kaicheng Li, Zhirong Liu, Yanxing Chen, Baorong Zhang

**Affiliations:** 1 Department of Neurology, The Second Affiliated Hospital, School of Medicine, Zhejiang University, Hangzhou, People’s Republic of China; 2 Department of Neurology, Affiliated Hangzhou First People’s Hospital, Zhejiang University School of Medicine, Hangzhou, People’s Republic of China; 3 Department of Geriatrics, The First Affiliated Hospital, School of Medicine, Zhejiang University, Hangzhou, People’s Republic of China

**Keywords:** Developmental biology, Neuroscience, Molecular medicine

## Abstract

Previous research has shown that exposure to volatile anesthetics can induce acute neuroinflammation and neuroapoptopsis in neonatal rodents and that these events can lead to cognitive dysfunction at later stages. Isoflurane and sevoflurane are two of the most popular anesthetics used in the field of pediatrics. However, the relative impact of these two anesthetics on the developing brain at distinct time points after the induction of anesthesia has not been compared. In the present study, we exposed 7-day-old mice to clinically equivalent doses of isoflurane (1.5%) and sevoflurane (2.5%) for 4 h and then investigated consequential changes in the brains of these mice at six different time points. We analyzed the levels of proteins that are directly related to neuroapoptosis, neuroinflammation, synaptic function, and memory, in the brains of neonatal mice. Exposure of neonatal mice to isoflurane and sevoflurane resulted in acute neuronal apoptosis. Our analysis observed significant levels of neuroinflammation and changes in the expression levels of proteins associated with both synaptic transmission and memory in mice from the isoflurane group but not the sevoflurane group. Our results therefore indicate that isoflurane and sevoflurane induce differential effects in the brains of neonatal mice.

## Introduction

Every year, millions of children are given general anesthesia so that they can undergo surgical procedures^1, 2^. A large retrospective study previously showed that children under the age of 4 years, who received repeated episodes of general anesthesia, were associated with an incidence of reading, writing, and mathematical learning disabilities, that was twice as high as those who had not been exposed to anesthesia^3^. Children exposed to anesthesia prior to 3 years-of-age were associated with a higher risk of deficits in language and abstract reasoning when they reached an age of 10 years^4, 5^. In addition, studies have revealed that the exposure of newborn animals to inhaled anesthetics can lead to a rapid and significant increase in acute neuroinflammation and neuroapoptosis, along with cognitive dysfunction at subsequent stages^6–9^. Therefore, serious concerns have been raised about the potential impacts of inhaled anesthetics on children. However, two large clinical trials, the General Anaesthesia and Awake-Regional Anaesthesia (GAS) trial and the Pediatric Anesthesia Neurodevelopment Assessment (PANDA) study, both showed that a single brief exposure to general anesthesia in early infancy did not affect neurodevelopmental outcomes during a given follow-up period^10, 11^. The GAS and PANDA studies involved neuropsychological tests that were performed at the ages of 5 and 10, respectively. However, cognitive and behavioral changes may not be obvious until later in life, as indicated by several preclinical studies^8^. Furthermore, the results derived from the GAS and PANDA studies cannot be extrapolated to conditions involving repeated or prolonged exposure to anesthesia. The potential neurotoxicity of anesthesia under these conditions has yet to be elucidated. It is likely that preclinical studies can provide a far better understanding of neurotoxicity relating to anesthesia.

Isoflurane and sevoflurane are two commonly used pediatric anesthetics. Therefore, it is of vital importance that we investigate their potential neurotoxicity. Both isoflurane and sevoflurane have been shown to induce neuroapoptosis in neonatal mice immediately after exposure^12^. Isoflurane has also been reported to induce greater levels of neuroapoptosis than sevoflurane in the brains of neonatal mice^13^. However, the short- and long-term effects of isoflurane and sevoflurane on apoptosis, inflammation, and synaptic protein levels, in the brains of newborn mice have yet to be investigated. Furthermore, very few studies have compared the effects of isoflurane and sevoflurane on the brains of neonatal mice at different time points following anesthesia, particularly with regards to differences over the long-term. Therefore, this study intended to investigate the relative effects of isoflurane and sevoflurane on apoptosis, neuroinflammation, and the synaptic levels of key proteins associated with memory, at different time points (from 0 h to 28 days) in the brains of postnatal day 7 mice mouse brain following 4 h of anesthesia.

## Methods

### Animals

All procedures were approved by the Institutional Animal Care and Use Committee of Zhejiang University and were conducted in accordance with the National Institutes of Health Guide for the Care and Use of Laboratory Animals guidelines for the ethical treatment of animals. Every effort was made to minimize the number of animals used in the experiments. Breeding pairs of C57BL6/J mice were obtained from the SLAC Laboratory Animal Company (Shanghai, China) and housed on a 12:12 light/dark cycle with ad libitum access to food and water. All experiments included both male and female pups; the ratio of males to females was 3:2.

### Exposure to anesthetics

On postnatal day 7 (P7), littermate mouse pups were randomly divided into three groups: (1) 4 h exposure to 100% oxygen (control); (2) 4 h exposure to 1.5% isoflurane in 100% oxygen; and (3) 4 h exposure of 2.5% sevoflurane in 100% oxygen. Groups of mice were exposed to the different treatments in specialized chambers. These doses represented 1 minimum alveolar concentration (MAC) of the two anesthetics, and had been validated in a pilot study, as described previously^9^. During anesthesia, we used a warming blanket to maintain rectal temperature at 38 ± 1 °C. Pups were allowed to wake up and recover on the warming blanket before they were returned to cages with their own mothers. None of the mice died during anesthesia. We sacrificed five mice from each group at 0 h, 4 h, 24 h, 7 days, 14 days, and 28 days, after exposure to anesthetic. We then removed the brains from these mice and snap-froze the cerebral cortices and hippocampi from the left hemisphere in dry ice; the samples were then stored at − 80 °C to await analysis. The right hemispheres were fixed with 4% paraformaldehyde, post-fixed overnight at 4 °C, and then dehydrated in 30% sucrose. Sagittal sections (30 µm thick) were then prepared on a cryostat and stored in glycol anti-freeze solution (glycerol and ethylene glycol in 0.1 M phosphate buffered saline [PBS]) at − 20 °C to await analysis.

### Western blotting

Frozen brain tissues were homogenized in ice-cold buffer with protease inhibitors containing 50 mM Tris–HCl (pH 7.4), 2.0 mM EGTA, 2 mM Na_3_VO_4_, 50 mM NaF, 20 mM β-glycerophosphate, 0.5 mM AEBSF, 10 μg/ml aprotinin, 10 μg/ml leupeptin, and 4 μg/ml pepstatin A. Extracted total protein was then separated by gel electrophoresis and transferred to a PVDF membrane (Millipore, Bedford, MA, USA). Following transfer, membranes were blocked in 5% skimmed milk in 0.1% tris-buffered saline/Tween-20 (TBST) for 1 h, and then incubated overnight with primary antibodies at 4 °C. The following morning, membranes were incubated with the appropriate horseradish peroxidase-conjugated secondary antibody for 1 h at room temperature. Positive antibody binding was then visualized by the ECL-PLUS system with enhanced chemiluminescent (ECL, Pierce, and Rockford, USA). Signal intensity was then analyzed with Image Lab (BIO-RAD, Hercules, CA).

### Enzyme-linked immunosorbent assays (ELISAs)

Frozen brain tissue was homogenized in ice-cold buffer with protease inhibitors. Brain homogenates were diluted (1:5) prior to ELISA and protein concentration was determined by bicinchoninic acid (BCA) protein assay (Pierce, Rockford, IL, USA). Levels of IL-1β, IL-6, and TNF-α, were then determined using specialized ELISA kits (Boster Biological Technology, Wuhan, China) in accordance with the manufacturer’s instructions.

### Immunofluorescence staining

Free floating sections of brain tissue were incubated for 20 min with 0.3% Triton X-100 at room temperature, washed in PBS, and blocked with 5% normal goat serum in PBS and 0.1% Triton X-100 for 30 min. Sections were then incubated overnight with primary antibodies at 4 °C. The following morning, sections were incubated with an appropriate secondary antibody for 1 h in darkness. We used normal goat serum in the absence of primary antibody as a negative control. After several washes with PBS, the sections were incubated with 4′,6-diamidino-2-phenylindole (DAPI) for 5 min and then visualized using an epifluorescence microscope.

### Antibodies and reagents

The primary antibodies used in this study are listed in Table 1. For western blotting, we purchased horseradish peroxide (HRP)-conjugated anti-mouse and anti-rabbit secondary antibodies from Invitrogen (Carlsbad, CA). For immunofluorescence, we acquired a goat anti-rabbit secondary antibody (Alexa Fluor 488, Life Technologies, Darmstadt, Germany) and a goat anti-mouse secondary antibody (Alexa Fluor 594, Life Technologies, Darmstadt, Germany). Other chemicals were purchased from Sigma (St. Louis, MO, USA).

**Table 1:** The primary antibodies used in this study.

Antibody	Source
Cleaved-caspase3	Cell Signaling Technology, Danvers, MA
Caspase-3	Cell Signaling Technology, Danvers, MA
Cleaved-PARP	Abcam, Cambridge, MA
PARP	Cell Signaling Technology, Danvers, MA
Caspase-9	MBL, Woburn, MA
Caspase-12	Cell Signaling Technology, Danvers, MA
IL-1β	Cell Signaling Technology, Danvers, MA
IL-6	Cell Signaling Technology, Danvers, MA
PSD-95	Cell Signaling Technology, Danvers, MA
Synaptophysin	Millipore, Temecula, CA, USA
Synapsin 1	Santa Cruz Biotechnology, Santa Cruz, CA
PKA-Cα	Cell Signaling Technology, Danvers, MA
p-CREB (Ser-133)	Cell Signaling Technology, Danvers, MA
CREB	Cell Signaling Technology, Danvers, MA
GFAP	Millipore, Temecula, CA, USA
Iba1	Wako Chemical, Richmond, VA, USA
β-actin	Hua’an, Hangzhou, China

### Statistical analysis

Data are presented as mean ± standard deviation (SD). Statistical analysis was performed using GraphPad Prism (GraphPad Software Inc., San Diego, CA, USA). All comparisons between mice in the anesthesia group and mice in the control group were carried out with the Student’s t-test. *P* values < 0.05 were considered to be statistically significant.

## Results

### Neuronal apoptosis in neonatal mice following exposure to isoflurane or sevoflurane

**Figure 1:**
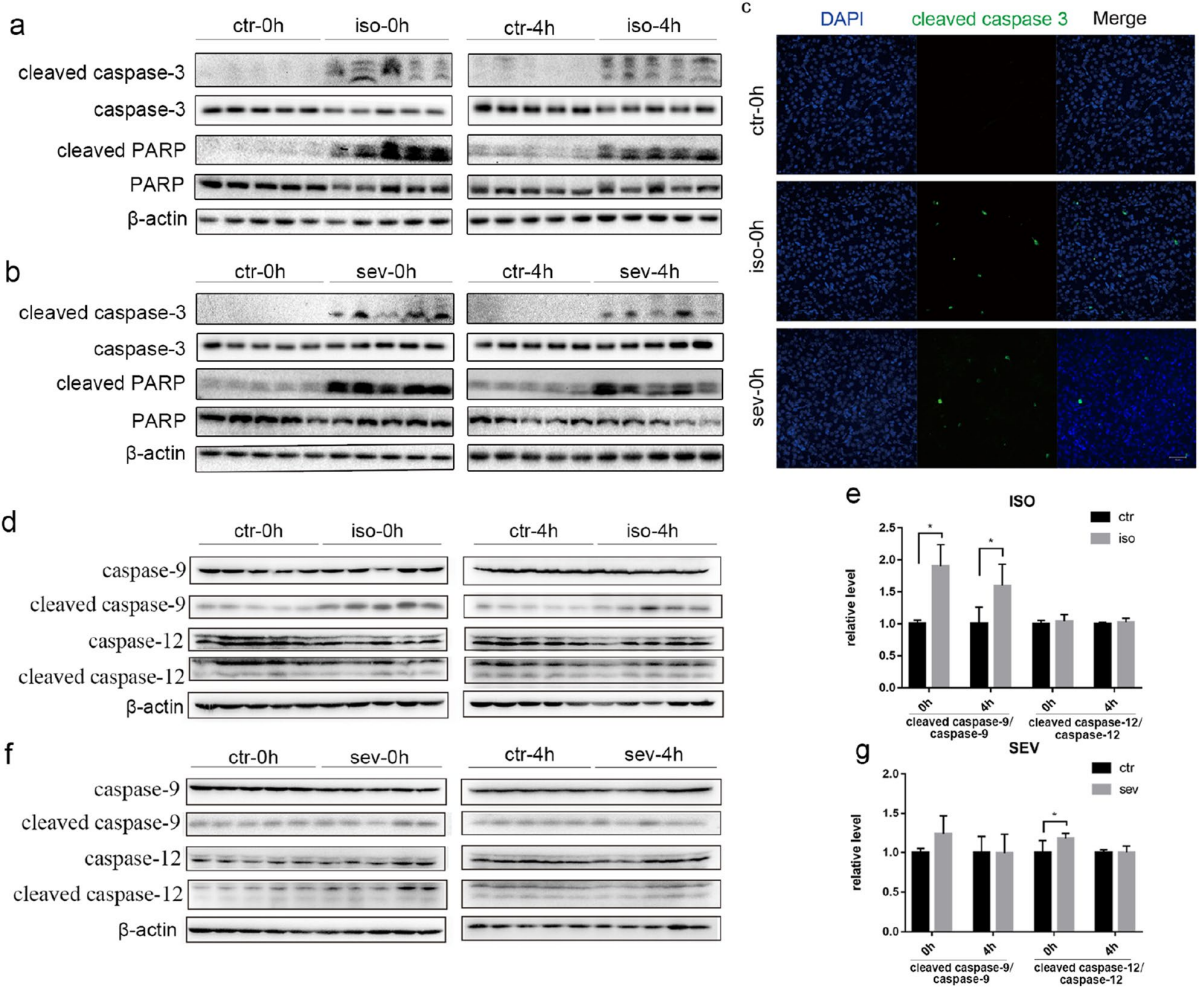
Neuronal Apoptosis in Neonatal Mice following Exposure to Isoflurane or Sevoflurane. (**a**, **b**, **d**, **f**) Brain homogenates from neonatal mice sacrificed at 0 h and 4 h after exposure to anesthesia were analyzed by western blotting using antibodies against cleaved caspase-3, caspase-3, cleaved PARP, PARP, cleaved caspase-9, caspase-9, cleaved caspase-12, caspase-12 and β-actin. Full-length blots are presented in Supplementary Fig. 3. (**c**) Representative immunofluorescence staining of the brain cortex from three groups immediately after treatment (scale bar, 50 μm). (**e**, **g**) Densitometric quantification of western blots after normalization to β-actin levels. Values are presented as mean ± SD. *P < 0.05 vs. Control. n = 5 per group. *Ctr* control, *iso* isoflurane, *sev* sevoflurane.

In order to evaluate the effect of isoflurane and sevoflurane on neuronal apoptosis, we investigated the activation of two markers of apoptosis, caspase-3 and poly ADP-ribose polymerase (PARP), in the brains of neonatal mice. Immediately after exposure to anesthetic for 4 h, we observed significantly increased expression levels of cleaved caspase-3 in mice from both the isoflurane and sevoflurane groups when compared with the control group (Fig. 1). The activation of caspase-3 lasted for at least 4 h. Similar changes were also observed for cleaved PARP in mice from both the isoflurane and sevoflurane groups (Fig. 1a,b). Immunofluorescence staining further confirmed that the number of activated caspase-3-positive cells was significantly increased in both of the anesthetic groups immediately after exposure (Fig. 1c). However, at 24 h, 7 days, 14 days, and 28 days, after exposure to anesthetic, we were unable to detect an apoptotic response in any of the groups, as evidenced by the absence of any positive signals for cleaved caspase-3 or cleaved PARP on western blotting.

To explore the possible mechanisms underlying the anesthetic-induced apoptosis, we examined the activation of caspase-9, which plays an important part in the mitochondrial pathway of apoptosis^14^. The level of cleaved caspase-9 was significantly higher than that of the control group at 0 h and 4 h after exposure to isoflurane (Fig. 1d,e), indicating the involvement of mitochondrial pathway in isoflurane-induced apoptosis. However, there were no significant differences in the level of active caspase-9 between the sevoflurane and control group (Fig. 1f,g). We also checked the activation of caspase-12, which is a major mediator of endoplasmic reticulum (ER) stress-induced apoptosis^15^. The level of cleaved caspase-12 was higher than that of the control group at 0 h after exposure to sevoflurane (Fig. 1f,g), implying the involvement of ER stress in sevoflurane-induced apoptosis. However, no differences in cleaved caspase-12 were observed between the isoflurane and control group (Fig. 1d,e). These results suggest different apoptotic pathways involved in anesthetic-induced apoptosis.

### Inflammatory responses in neonatal mice following exposure to isoflurane or sevoflurane

**Figure 2:**
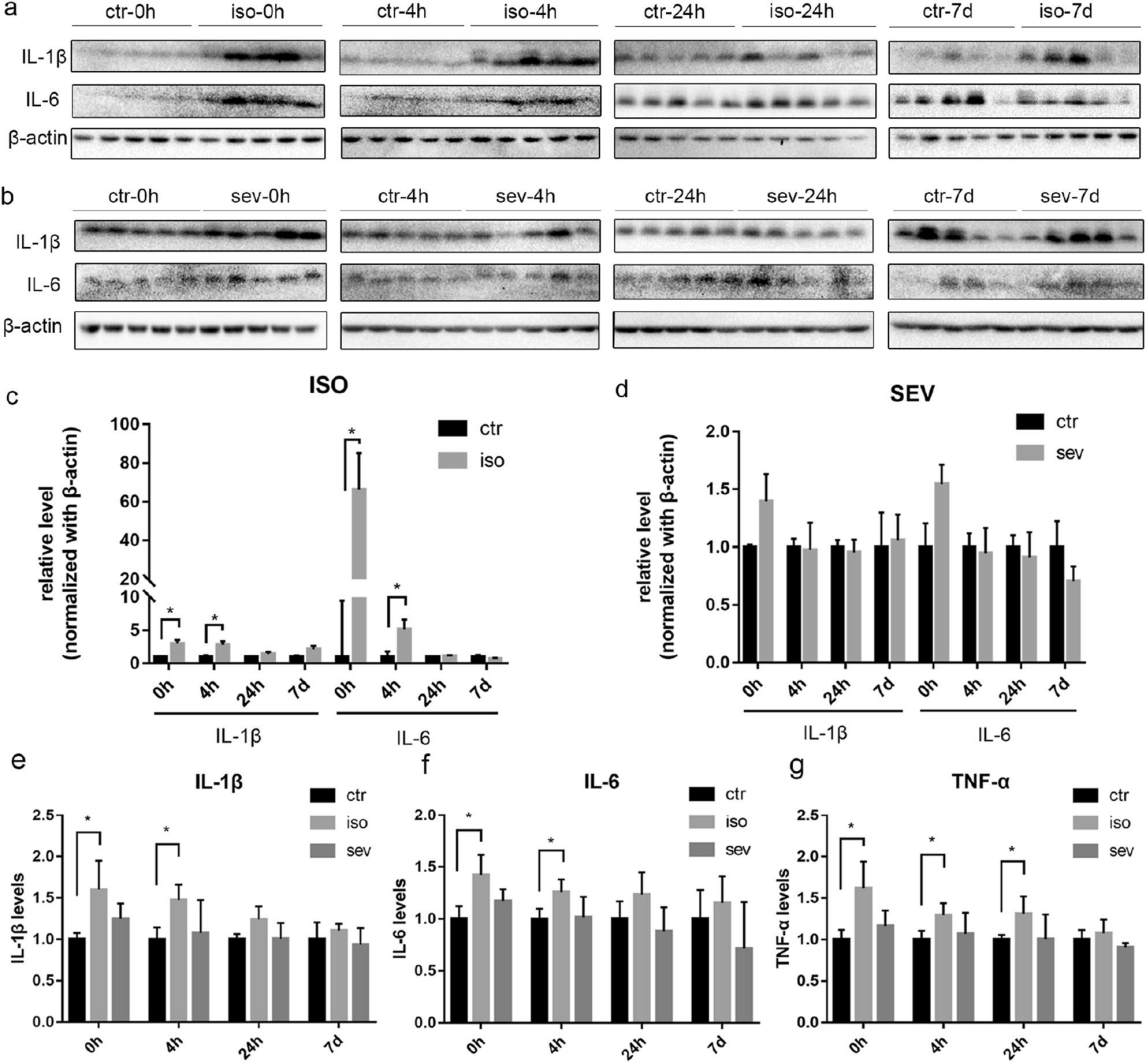
Inflammatory Responses in Neonatal Mice following Exposure to Isoflurane or Sevoflurane. (**a**, **b**) Brain homogenates from neonatal mice sacrificed at 0 h, 4 h, 24 h, and 7 days, after exposure to anesthesia were analyzed by western blotting with antibodies against IL-1β, IL-6, and β-actin. Full-length blots are presented in Supplementary Fig. 4. (**c**, **d**) Densitometric quantification of blots after normalization to β-actin level. The levels of IL-1β (**e**), IL-6 (**f**), and TNF-α (**g**), were investigated by ELISA. The relative levels of these proteins are shown after normalization to the control. Values are presented as mean ± SD. *P < 0.05 vs. Control. n = 5 per group. *Ctr* control, *iso* isoflurane, *sev* sevoflurane.

In order to investigate the effects of isoflurane and sevoflurane on neuroinflammation in neonatal mice, we determined the expression levels of several proinflammatory factors (IL-1β, IL-6, and TNF-α). Western blotting and ELISA both showed that the levels of IL-1β and IL-6 levels were significantly higher than the control group at 0 h and 4 h after exposure to isoflurane; these levels had returned to the normal range when tested at 24 h and 7 days after exposure. The levels of TNF-α were significantly higher than those of the control group at 0 h, 4 h, and 24 h, after exposure to isoflurane; levels of TNF-α had returned to the normal range when tested 7 days after exposure to isoflurane (Fig. 2). However, we failed to detect any significant increases with regards to the levels of IL-1β, IL-6, or TNF-α, at any time points after exposure to sevoflurane. We also investigated the activation of glial fibrillary acidic protein (GFAP) and ionized calcium binding adapter molecule 1 (Iba1); these are known markers of astroglia and microglia, respectively. No significant differences were detected between the isoflurane or sevoflurane groups when compared with the control group (Supplementary Fig. 1, 2).

### Differential changes in the synaptic proteins of neonatal mice exposed to isoflurane or sevoflurane

**Figure 3:**
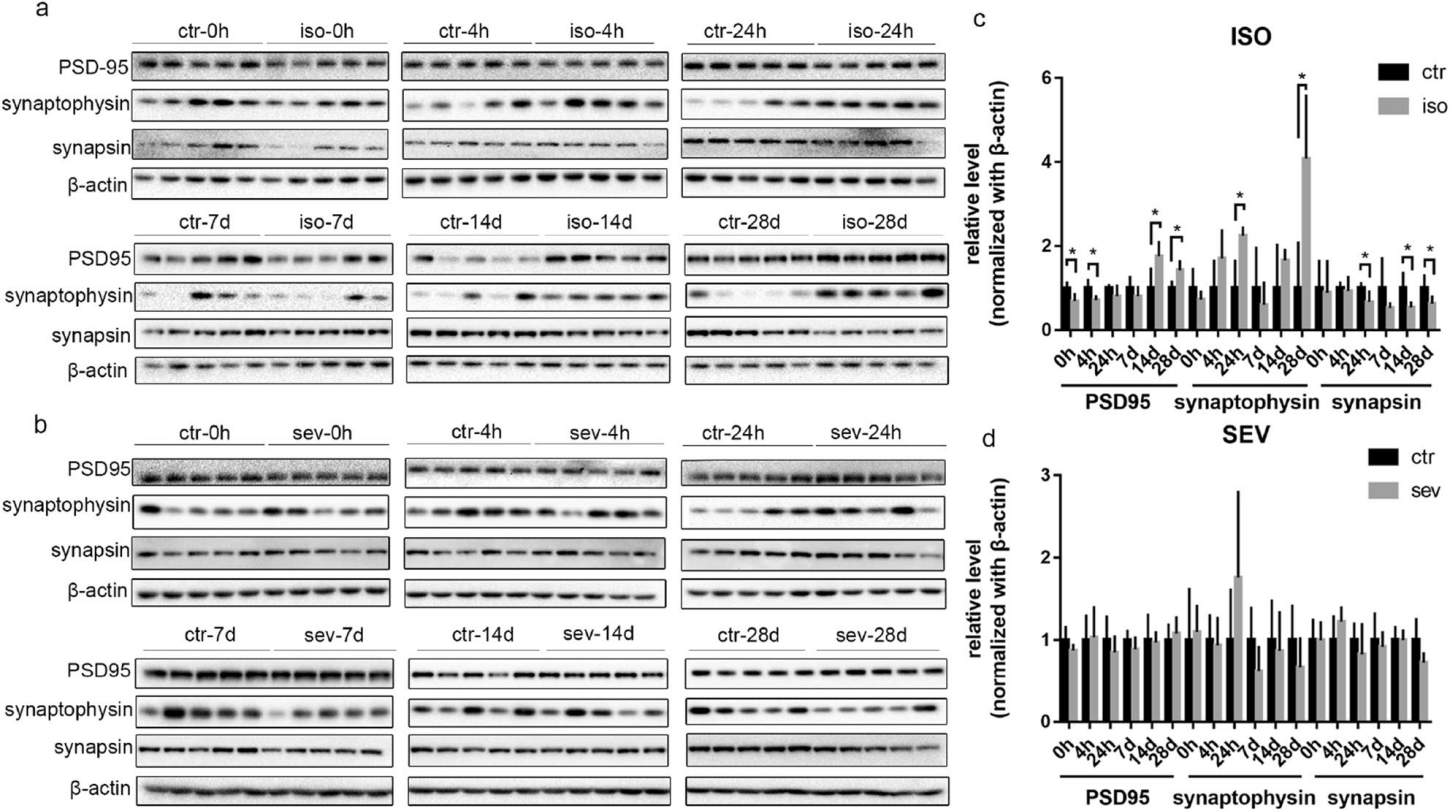
Differential changes in the Levels of Key Synaptic Proteins in Neonatal Mice Exposed to Isoflurane or Sevoflurane. (**a**, **b**) Brain homogenates from neonatal mice sacrificed at 0 h, 4 h, 24 h, 7 days, 14 days, and 28 days, after exposure to anesthesia were analyzed by western blotting using antibodies against PSD-95, synaptophysin, synapsin, and β-actin. Full-length blots are presented in Supplementary Fig. 5. (**c**, **d**) Densitometric quantification of western blots after normalization to β-actin levels. Values are presented as mean ± SD. *P < 0.05 vs. Control. n = 5 per group. *Ctr* control, *iso* isoflurane, *sev* sevoflurane.

**Figure 4:**
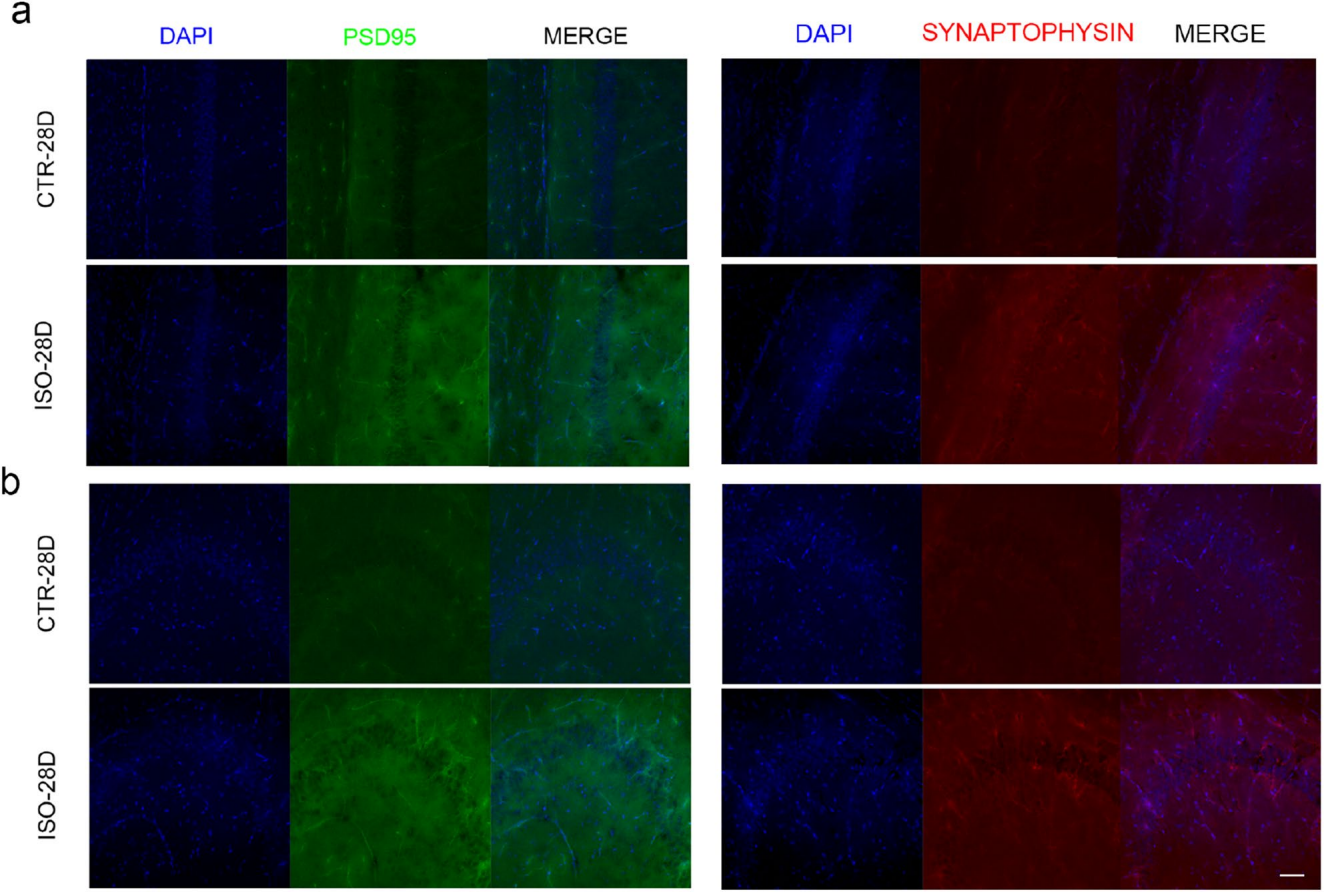
Immunofluorescence staining of PSD-95 and Synaptophysin in Neonatal Mice following Exposure to Isoflurane or Sevoflurane. Representative immunofluorescence staining for PSD-95 (green) and synaptophysin (red) in CA1 (**a**) and CA3 (**b**) of the hippocampus from the two groups on day 28 after exposure to anesthetics (scale bar, 50 μm). The density of PSD-95 and synaptophysin immunostaining in the hippocampus was greater in the isoflurane group than in the control group. Cell nuclei were stained with DAPI (blue). n = 5 per group. *Ctr* control, *iso* isoflurane, *sev* sevoflurane.

In order to investigate the effects of isoflurane and sevoflurane on the expression of synaptic proteins, we determined the levels of PSD-95, synaptophysin, and synapsin at several time points (0 h, 4 h, 24 h, 7 days, 14 days, and 28 days) after 4 h of anesthetic exposure; these are post-, pre-, and pre-synaptic markers, respectively. The levels of PSD-95 were significantly reduced at 0 h and 4 h after isoflurane exposure as compared to the control group, but were increased at 14 days and 28 days (Fig. 3). After exposure to isoflurane, the levels of synaptophysin remained unchanged at 0 h, 4 h, 7 days, and 14 days, but were higher than the control group at 24 h and 28 days after exposure. In contrast, the levels of synapsin were downregulated when tested 14 days and 28 days after exposure to isoflurane. The increased levels of PSD-95 and synaptophysin on day 28 after exposure to isoflurane were also confirmed by immunofluorescence analysis (Fig. 4). However, no significant changes were identified in the sevoflurane group with respect to the levels of any of the synaptic proteins tested.

### The expression of memory-associated proteins in the brains of neonatal mice exposed to isoflurane or sevoflurane

**Figure 5:**
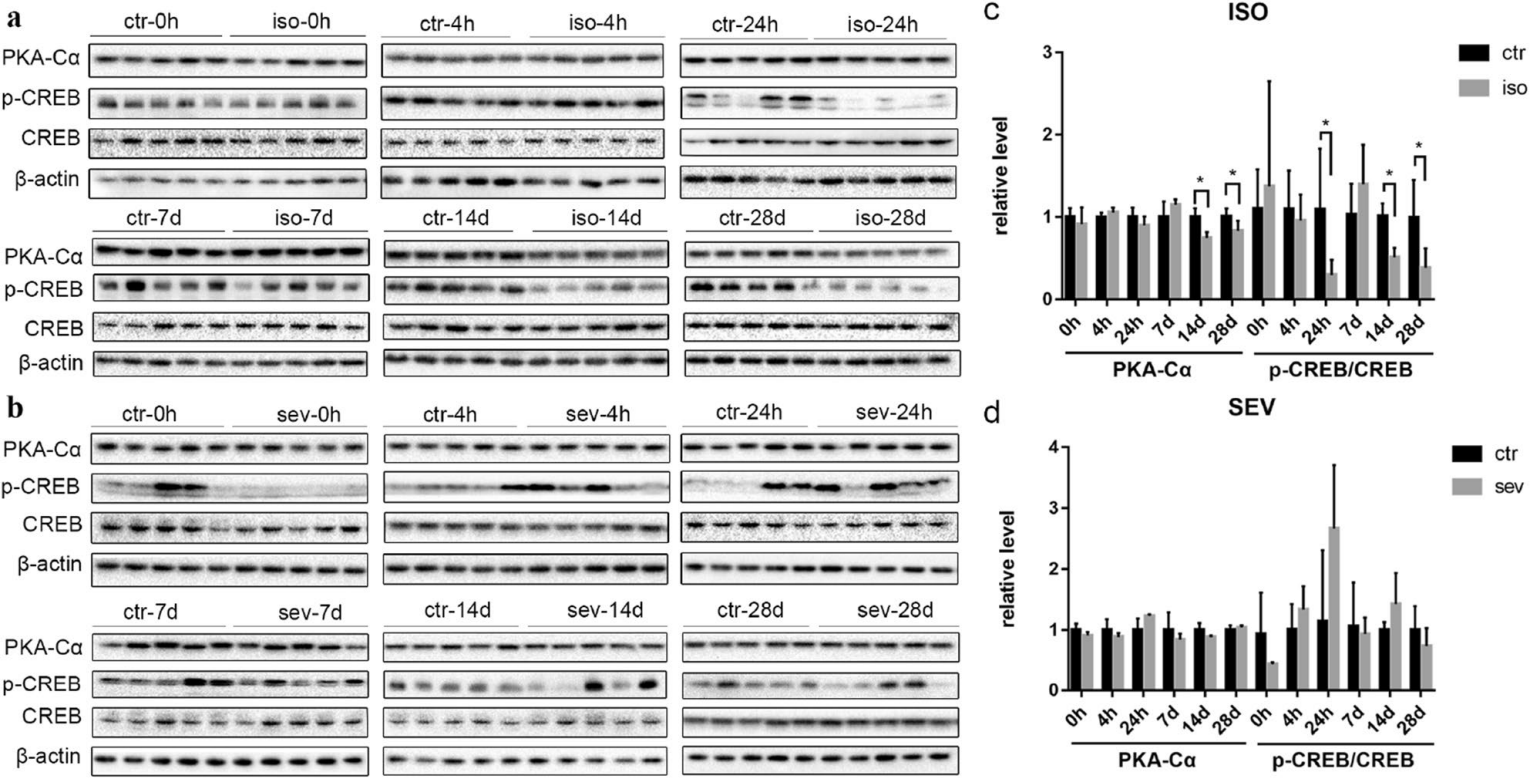
The Expression of Memory-Associated Proteins in Neonatal Mice Exposed to Isoflurane or Sevoflurane. (**a**, **b**) Brain homogenates from neonatal mice sacrificed at 0 h, 4 h, 24 h, 7 days, 14 days, and 28 days, after exposure to anesthetics were analyzed by western blotting using antibodies against PKA-Cα, p-CREB, CREB, and β-actin. Full-length blots are presented in Supplementary Fig. 6. (**c**, **d**) Densitometric quantification of western blots after normalization to β-actin levels. Values are presented as mean ± SD. * *P* < 0.05 vs. Control. n = 5 per group. *Ctr* control, *iso* isoflurane, *sev* sevoflurane.

In order to investigate the effects of both volatile anesthetics on memory, we investigated the expression levels of PKA-Cα, the cAMP responsive element binding protein (CREB), and p-CREB, the activated form of CREB. There were no significant differences in the levels of PKA-Cα or p-CREB when compared between the isoflurane group and the control group (Fig. 5) at 0 h, 4 h, 24 h, or 7 days, after anesthesia. However, the levels of PKA-Cα in the isoflurane group were significantly lower than the control group on days 14 and 28 after exposure; these observations were also consistent with the reduced ratio of p-CREB to CREB at these two time points. In contrast, exposure to sevoflurane did not have any significant effect on the levels of any of these proteins at any of the time points.

## Discussion

For many years, concerns have been raised over the potential for anesthetics to induce neurological abnormalities. Some recent clinical studies have indicated that a single, brief exposure to general anesthesia during infancy might not necessarily lead to neurodevelopmental disabilities^10, 11^. However, lengthy or repeated exposure to anesthetics may have a deleterious impact on brain development^16^. Preclinical studies could provide us with valuable mechanistic understanding of the neurotoxicity associated with anesthesia. In the present study, we investigated the effects of isoflurane and sevoflurane on neuroapoptopsis, neuroinflammation, synaptic proteins, and memory-associated proteins, at distinct time points after anesthesia. We observed a significant increase in neuroapoptosis at 0 h and 4 h after exposure to both isoflurane and sevoflurane. There was also an enhancement in neuroinflammation at 0 h and 4 h post-anesthesia in the isoflurane group but not in the sevoflurane group. We also observed significant changes in the levels of synaptic proteins at different time points in the isoflurane group; however, in the sevoflurane group, we only observed a reduction in the levels on day 28 post-anesthesia. Furthermore, the expression levels of p-CREB and PKA-Cα were reduced at 14 days and 28 days post-anesthesia in the isoflurane group but not in the sevoflurane group.

Neuronal apoptosis plays an indispensable role in brain development; however, the over-activation of this process could affect neural development and thus contribute to behavioral changes later in life. Several preclinical studies have consistently reported that inhaled anesthetics are associated with acute neuroapoptosis in the developing brain^12, 17^. In order to investigate whether isoflurane and sevoflurane exert differential impacts on neuroapoptosis, we determined the levels of several apoptosis markers at six different time points after anesthesia. We found that caspase-3, the key biomarker of apoptosis, was upregulated at 0 h and 4 h after exposure to both isoflurane and sevoflurane; these findings concurred with those published previously^12, 17^. Cleaved PARP is the main cleavage target of caspase-3. Consistently, we observed the upregulation of PARP at 0 h and 4 h after exposure to both isoflurane and sevoflurane. However, the upregulation of these two markers of apoptosis was not, detected by western blotting at 24 h after anesthesia, thus indicating that neuroapoptosis only occurred during the acute phase after anesthesia and subsequently returned to normal levels. Several previous preclinical studies have also reported that the inhalation of these two anesthetics can lead to neuronal apoptosis^12, 17^. Some studies have also indicated that isoflurane might induce more robust apoptosis than sevoflurane^13, 17^. In the current study, we did not compare the specific extent of neuroapoptosis; however, it is clear that apoptosis is a common phenomenon when neonatal mice are exposed to isoflurane and sevoflurane. Additionally, we found the activation of caspase-9 by isoflurane but not sevoflurane, indicating the involvement of mitochondrial apoptotic pathway in isoflurane-induced apoptosis. Procaspase-9 combines with cytochrome-c, which is released from the mitochondria, to form an apoptosome, resulting in the cleavage and activation of caspase-9. Cleaved caspase-9 then induced the activation of caspase-3, leading to apoptosis^14^. On the other hand, the activation of caspase-12 was observed in neonatal mice exposed to sevoflurane but not isoflurane. Excessive ER stress can trigger apoptosis through the activation of caspase-12, which is localized to ER, and is specifically activated by ER stress^15^. Active caspase-12 can activate caspase-3, independently of the activation of caspase-9, leading to apoptosis^18^. On the other hand, active caspase-12 can also trigger the activation of caspase-9, leading to the activation of caspase-3^15^. Here, we found the activation of caspase-12 but not caspase-9 in mice exposed to sevoflurane, suggesting a caspase-9-independent manner in sevoflurane-induced ER stress. Collectively, the current study indicates that isoflurane and sevoflurane induced apoptosis through different mechanisms.

Neuroinflammation during early brain development is associated with a wide range of neuropsychiatric diseases, including autism, Alzheimer’s disease, and schizophrenia^19^. IL-6, IL-1β, and TNF-α, are the major proinflammatory cytokines and are known to influence neuronal differentiation, proliferation, and neurogenesis^20^. IL-6 has also been shown to be associated with spatial learning and memory impairment^21^. Therefore, we investigated the expression of IL-6, IL-1β, and TNF-α, in neonatal mice exposed to anesthetic. Following exposure to isoflurane, we found that mice exhibited transient increases in the levels of IL-1β, IL-6, and TNF-α. A previous study also showed that exposure to 1.8% isoflurane for 4 h induced an increase in the expression of proinflammatory cytokines (TNF-α and IL-1β) in the brains of P7 rats^22^. Surprisingly, we did not observe an increase in the levels of these proinflammatory cytokines at any time points in neonatal mice exposed to sevoflurane. Previous studies also found that a single exposure of neonatal mice to 3% sevoflurane for 2 h or 6 h did not increase the levels of IL-6 or/and TNF-α in the brain^23, 24^. On the other hand, however, repeated exposure (3% sevoflurane, 2 h daily for 3 days), led to a notable increase in the levels of IL-6, IL-1β, and TNF-α, in the brains of neonatal mice immediately after anesthesia^23, 25, 26^. This discrepancy may be related to the different doses of anesthetics used, thus indicating that repeated episodes of anesthesia are far more neurotoxic and that the neurotoxicity of sevoflurane in neonatal mice is both dose- and time-dependent. Collectively, our study indicates that isoflurane can cause acute neuroinflammation in the brain of neonatal mice while sevoflurane does not.

Synapsin and synaptophysin are synaptic vesicle proteins. Synapsin regulates the exocytosis of synaptic vesicles while synaptophysin regulates the endocytosis of synaptic vesicles. During synaptic development, these two proteins play key roles in the maturation of presynaptic function^27^. PSD-95 is important for the conversion of synaptic function from a silent state to a fully functional state^28^ and is also a good marker for post-synaptic development. Therefore, to investigate the effects of isoflurane and sevoflurane on synaptic function, we investigated the expression of synapsin, synaptophysin, and PSD-95, in neonatal mice. We found that the expression levels of PSD-95 and synaptophysin were increased in the brains of neonatal mice exposed to isoflurane, while the levels of synapsin were reduced. We did not observe any changes in the expression of synaptic proteins when neonatal mice were exposed to sevoflurane. Although a previous study reported the reduced expression of PSD-95 at P8 and P31 after multiple exposures to sevoflurane at P8^29^, our current study suggests that a single exposure to sevoflurane during the early development may have little effect on the expression of synaptic proteins.

As an important nuclear protein, CREB is widely expressed in the hippocampus and cortex of rodents and plays an important role in synaptic formation, neurogenesis, learning, and memory^30^. The activation of CREB can lead to the transcription of gene products involved in synaptic plasticity and cognitive function, such as brain derived neurotrophic factor (BDNF), thereby enhancing memory formation. CREB is activated via the phosphorylation of Ser-133 by PKA-Cα^31^. In the present study, we observed reduced levels of PKA-Cα and p-CREB on days 14 and 28 after exposure to isoflurane. This observation is consistent with a previous study that reported the inactivation of CREB by isoflurane, thus resulting in the loss of dendritic outgrowth and the reduced expression of proteins that are essential for memory and cognitive functions in the brains of P7 mice, including BDNF and c-fos^32^. In contrast, in our present study, we found that the levels of PKA-Cα and p-CREB remained unchanged following exposure to sevoflurane. These results suggest that a single exposure to isoflurane, but not sevoflurane, may induce the inactivation of CREB in the developing brain, and that this process might underlie the cognitive impairments that are associated with anesthesia.

Several possible mechanisms have been proposed to explain the neurotoxicity induced by volatile anesthetics. For example, previous reports have suggested that volatile anesthetics could induce abnormal calcium release from the endoplasmic reticulum via the excessive activation of IP_3_ receptors, thus leading to cell apoptosis. Isoflurane is known to exhibit a greater potency to disrupt intracellular calcium homeostasis than sevoflurane^33–35^. The anesthetic-induced suppression of synaptic signaling can also lead to apoptosis by directly activating the intrinsic apoptotic cascade^36^. Furthermore, anesthetics could block normal neurotransmission by binding to gamma-aminobutyric acid (GABA_A_) receptors, thus leading to synaptic deprivation. Alterations in GABA_A_ signaling could also lead to pro-inflammatory effects of anesthesia in the developmental brain, which might also trigger the extrinsic apoptotic cascade^36^. It is possible that isoflurane and sevoflurane have differential potentials for initiating these cascades. In the current study, we detected the activation of caspase-9 in isoflurane exposed neonatal mice, indicating the involvement of mitochondrial apoptotic pathway. It has been shown that aberrant activation of caspase-9 led to dystrophy of neurites and synaptic-memory deficits, which could be rescued by caspase-9 inhibition^37^. Therefore, caspase-9 mediated downstream events might also attribute to the changes to synaptic proteins and memory-associated proteins induced by isoflurane. However, further studies are needed to elucidate the specific mechanisms that underline these effects.

The present study has several limitations that should be considered. First, we did not investigate behavioral changes that may have been induced by the two different anesthetics. Cognitive and behavioral abnormalities have been widely investigated by previous studies. Given that the main aim of the current study was to compare the biochemical effects of isoflurane and sevoflurane exposure at different time points, we did not perform any behavioral tests. Second, it has been suggested that translating the results of preclinical studies that involve the mouse model to humans might be misleading. A recent study, employing single-nucleus RNA sequencing to classify cell types in the brains of humans and mice found that two thirds of the 9748 genes evaluated were differentially expressed between these two species^38^. However, it is obvious that ethical considerations create a major limitation with regards to carrying out research on human newborns. We firmly believe that preclinical studies involving mice can still yield valuable information relating to the mechanisms underlying neurotoxicity induced by exposure to anesthetics.

## Conclusions

In conclusion, we found both isoflurane and sevoflurane can induce neuroapoptosis through different pathways in neonatal mice. However, only mice exposed to isoflurane showed significant levels of neuroinflammation and the abnormal expression of proteins associated with synaptic function and memory. Our study provides clear evidence for the differential effects of isoflurane and sevoflurane on the brains of neonatal mice. Collectively, our findings enhance our understanding of the potential problems associated with the use of these volatile anesthetics in pediatric medicine.

## Supplementary information

Supplementary Figures.

## Data Availability

All relevant data for this study are included in the manuscript/supplementary files.

## Abbreviations

Cleaved-PARP: Cleaved poly ADP-ribose polymerase
PARP: Poly-ADP-ribose polymerase
IL-6: Interleukin-6
IL-1β: Interleukin-1 beta
TNF-α: Tumor necrosis factor alpha
PSD-95: Post synaptic density protein 95
CREB: CAMP response element binding protein
p-CREB: Phosphorylated cAMP response element binding protein
PKA-Cα: CAMP-dependent protein kinase A catalytic subunits alpha
MAC: Minimum alveolar concentration
ELISA: Enzyme-linked immunosorbent assay
SD: Standard deviation
GFAP: Glial fibrillary acidic protein
Iba1: Ionized calcium binding adapter molecule 1
BDNF: Brain-derived neurotrophic factor
GABA: G-aminobutyric acid
ctr: Control
iso: Isoflurane
sev: Sevoflurane
