# Supplementary material for: The differential effects of isoflurane and sevoflurane on neonatal mice
Source: Sci Rep. 2020 Nov 9;10:19345. doi: 10.1038/s41598-020-76147-6 (PMC7652873; doi:10.1038/s41598-020-76147-6)
Supplement: Supplementary file 1 — Supplementary Figures. [file 41598_2020_76147_MOESM1_ESM.pdf]

The differential effects of isoflurane and sevoflurane on neonatal mice

Shuai Zhao, PhD[1#], Ziqi Fan, MD[1#], Jing Hu, MD[1], Yueli Zhu, MD[1], Caixiu Lin, MD[1,2], Ting Shen, PhD[1], Zheyu Li, MD[1], Kaicheng Li, PhD[1], Zhirong Liu, MD, PhD[1], Yanxing Chen, MD, PhD[1*], Baorong Zhang, MD[1*]

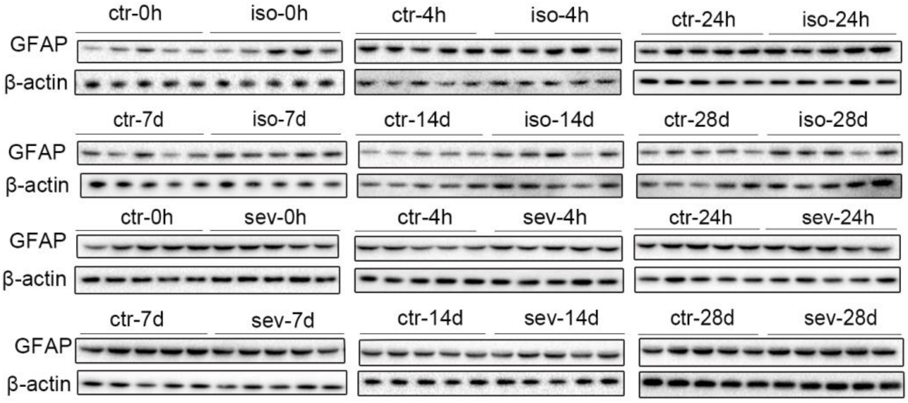

Supplementary Figure 1. Effects of Isoflurane and Sevoflurane on Astroglial Markers. Brain homogenates from neonatal mice sacrificed at 0h, 4h, 24h, 7d, 14d and 28d after anesthesia exposure were analyzed by Western blotting developed with antibodies against GFAP and β-actin. Full-length blots are presented in Supplementary Figure 7. n=5 per group. Ctr (control), iso (isoflurane), sev (sevoflurane).

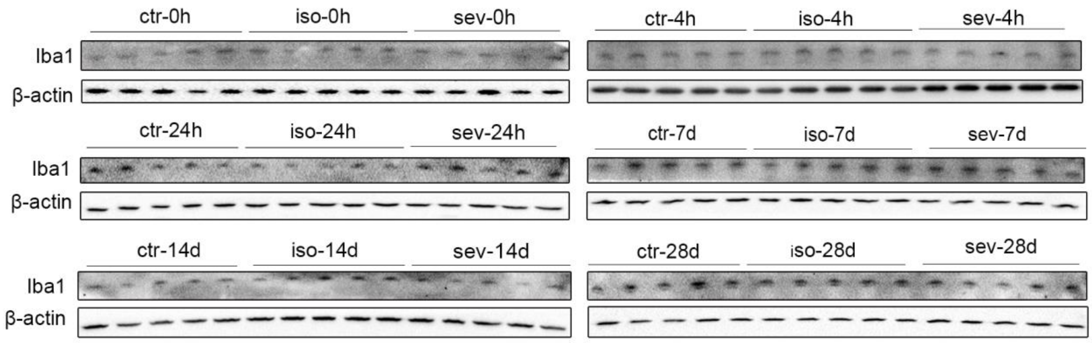

Supplementary Figure 2. Effects of Isoflurane and Sevoflurane on Microglial Markers. Brain homogenates from neonatal mice sacrificed at 0h, 4h, 24h, 7d, 14d and 28d after anesthesia exposure were analyzed by Western blotting developed with antibodies against Iba1 and β-actin. Full-length blots are presented in Supplementary Figure 8. n=5 per group. Ctr (control), iso (isoflurane), sev (sevoflurane).

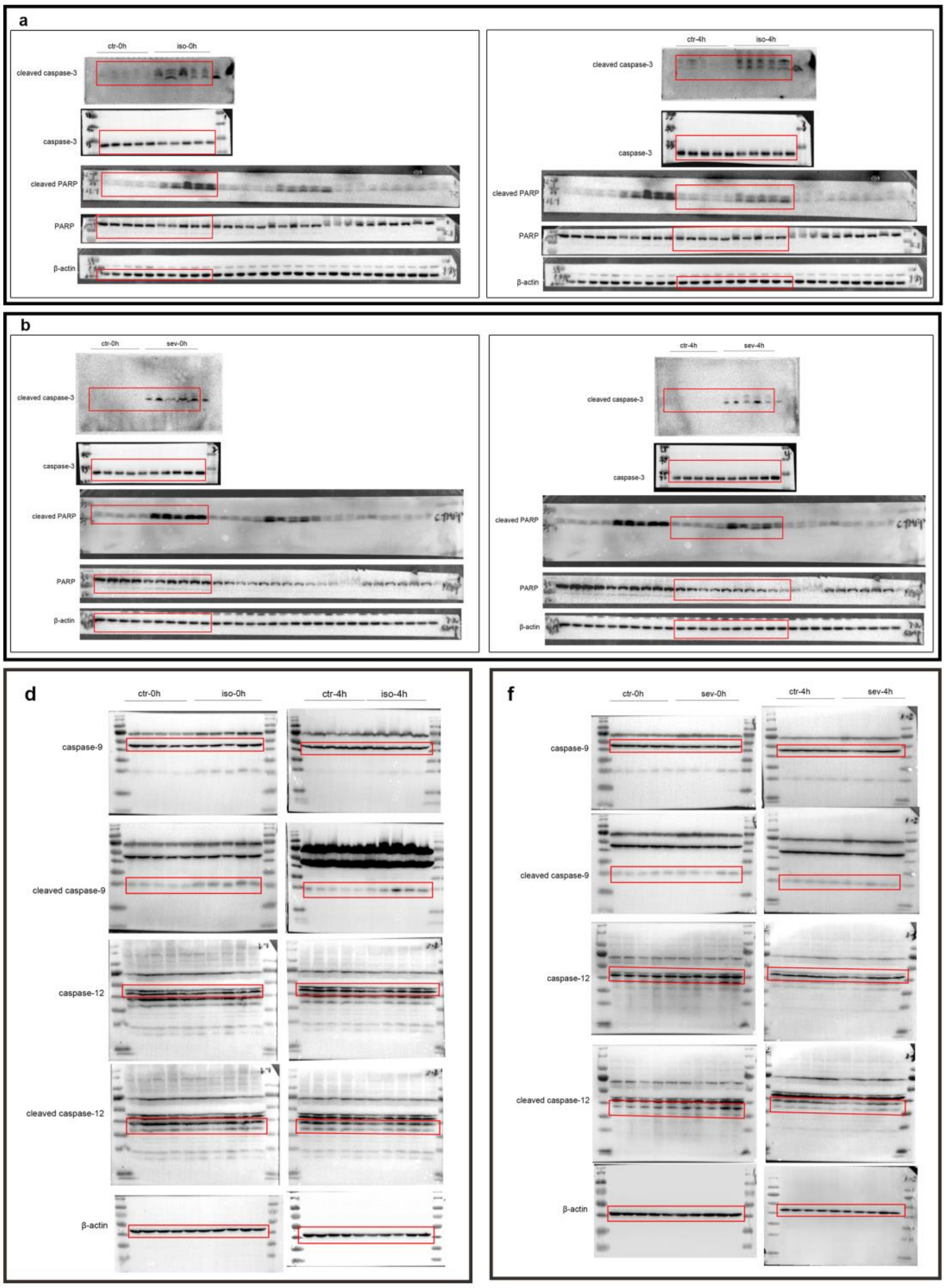

Supplementary Figure 3. Original, unprocessed blots of Fig. 1a (a), Fig. 1b (b), Fig. 1d (d) and Fig. 1f (f).

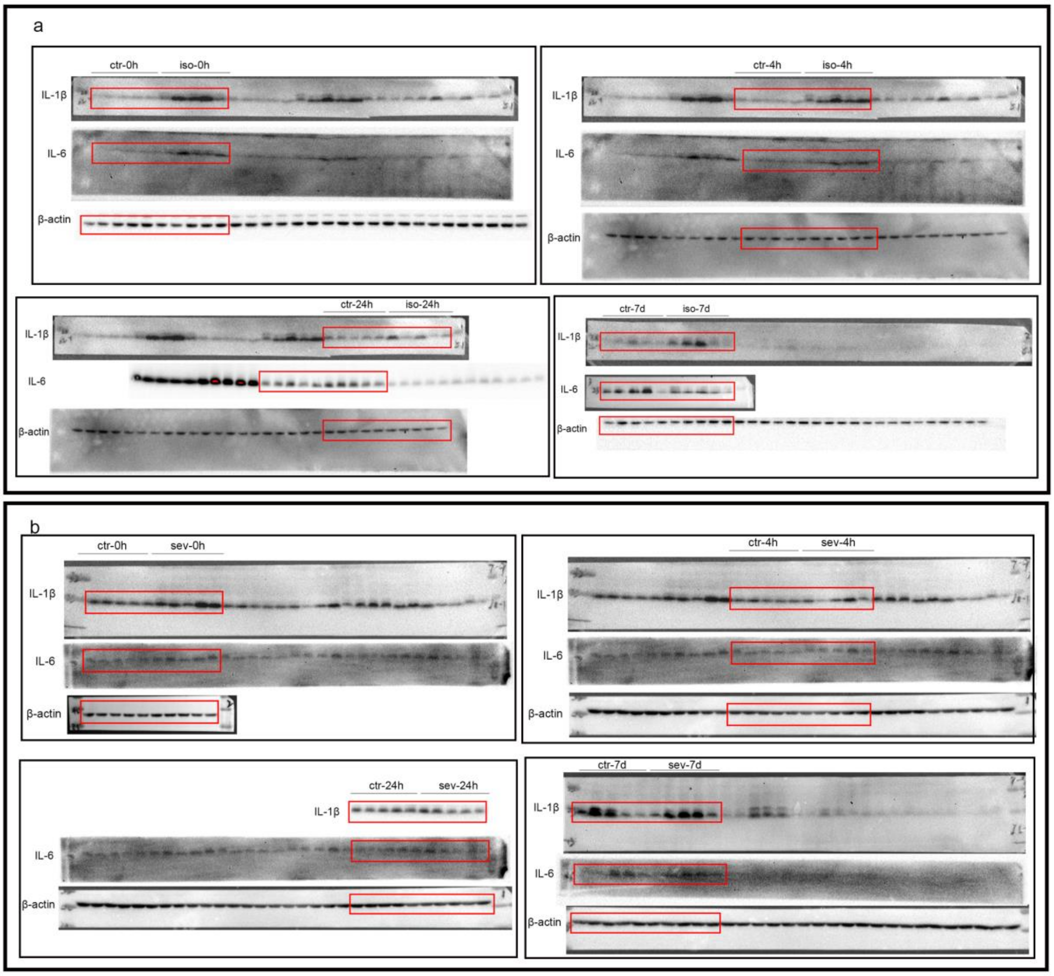

Supplementary Figure 4. Original, unprocessed blots of Fig. 2a (a) and Fig. 2b (b)

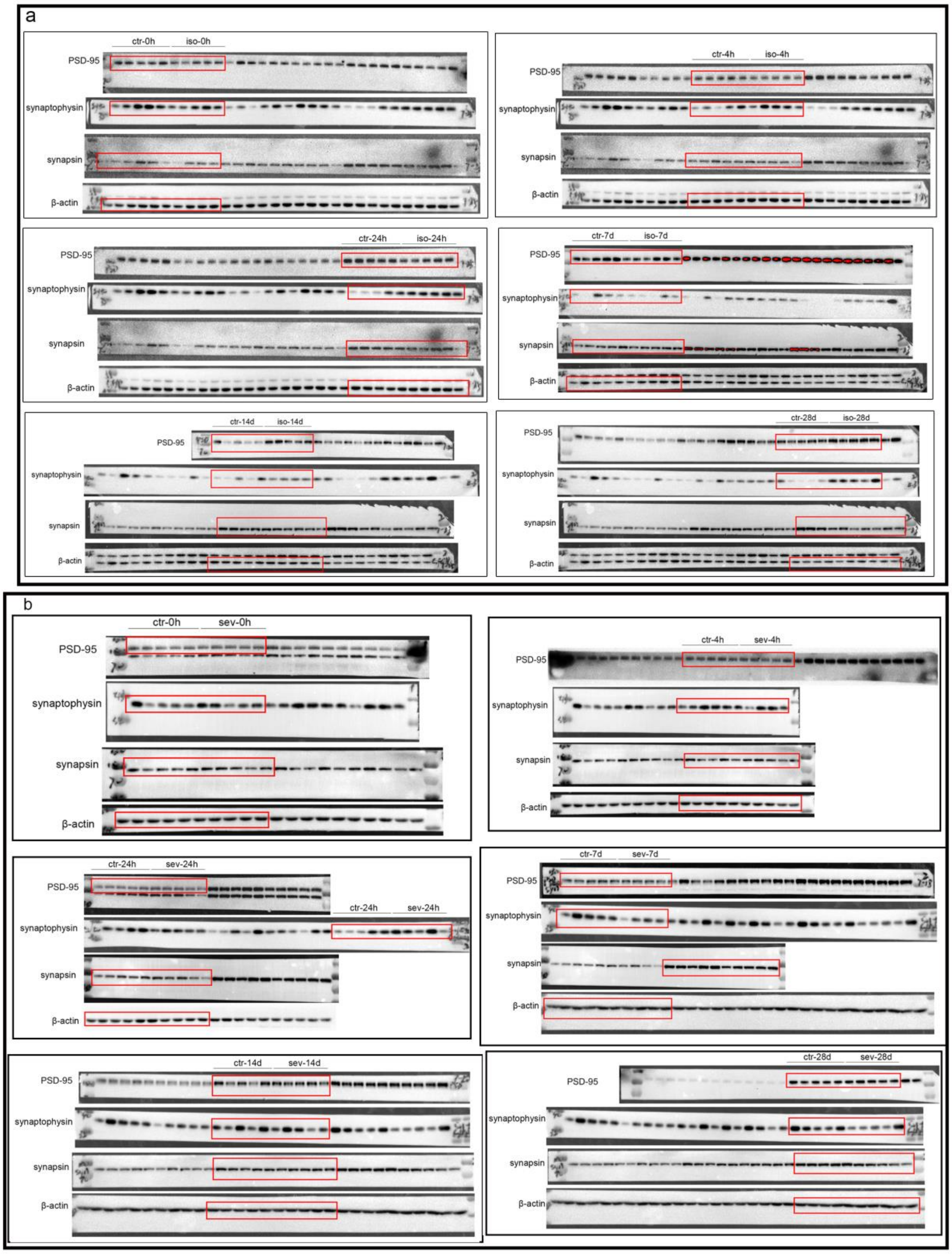

Supplementary Figure 5. Original, unprocessed blots of Fig. 3a (a) and Fig. 3b (b)

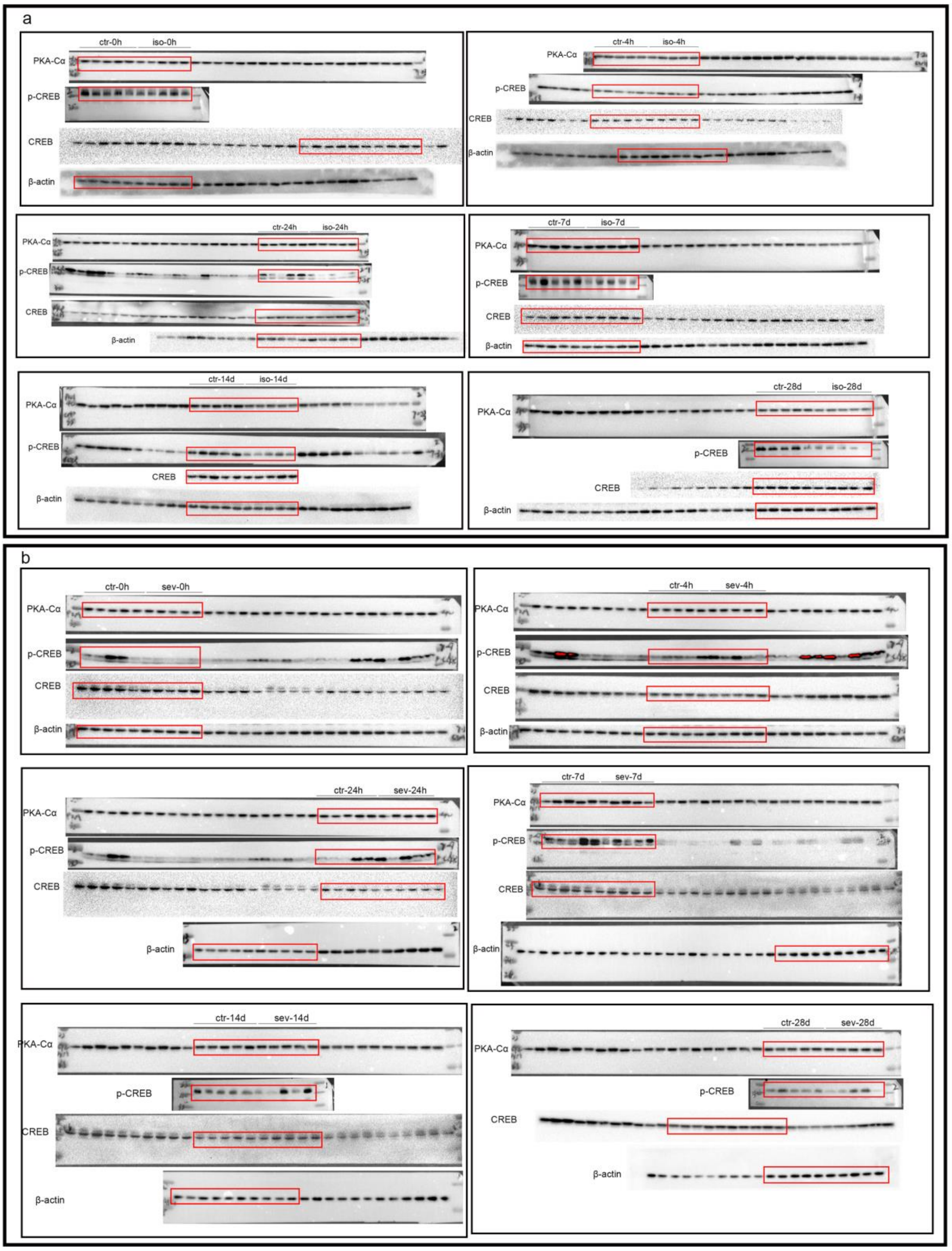

Supplementary Figure 6. Original, unprocessed blots of Fig. 5a (a) and Fig. 5b (b)

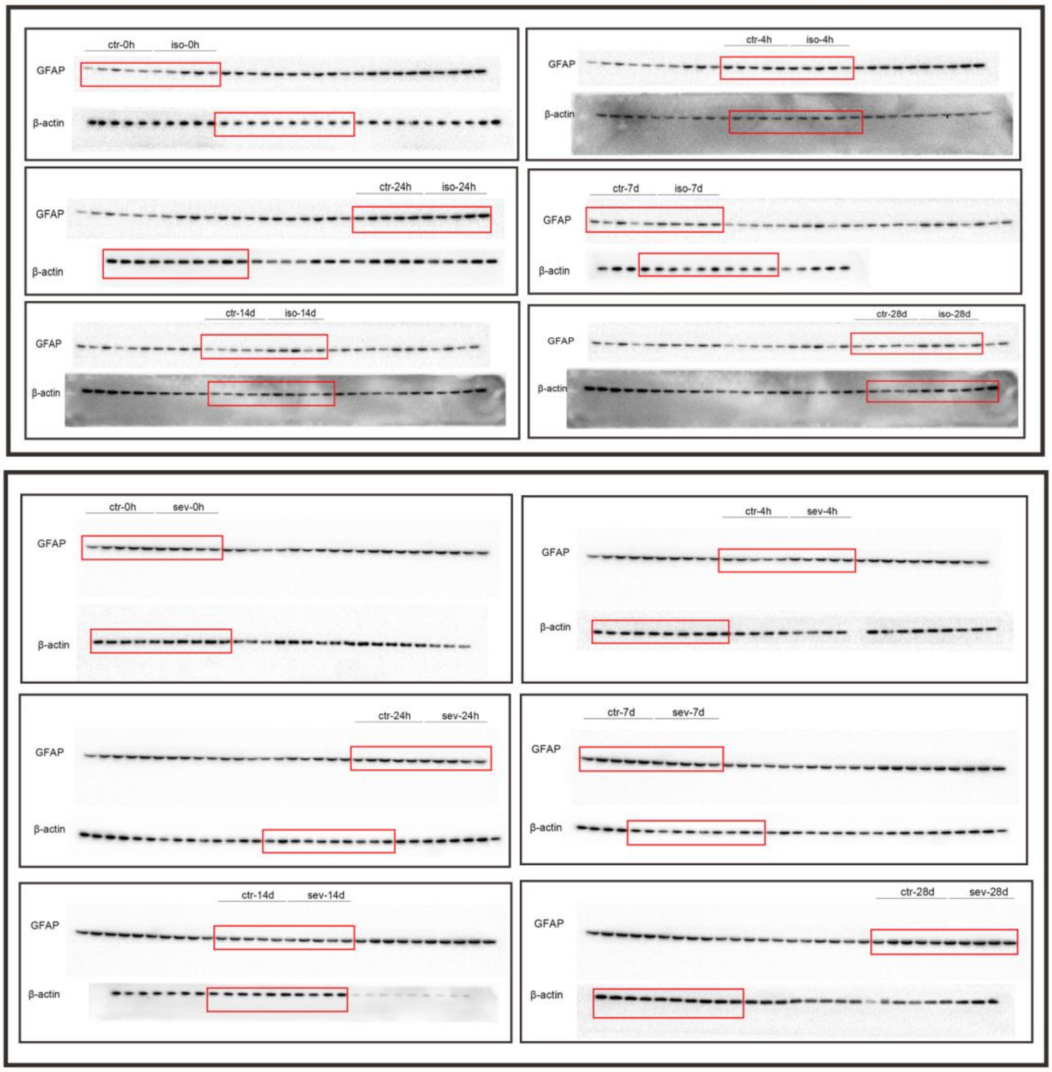

Supplementary Figure 7. Original, unprocessed blots of Supplementary Fig. 1

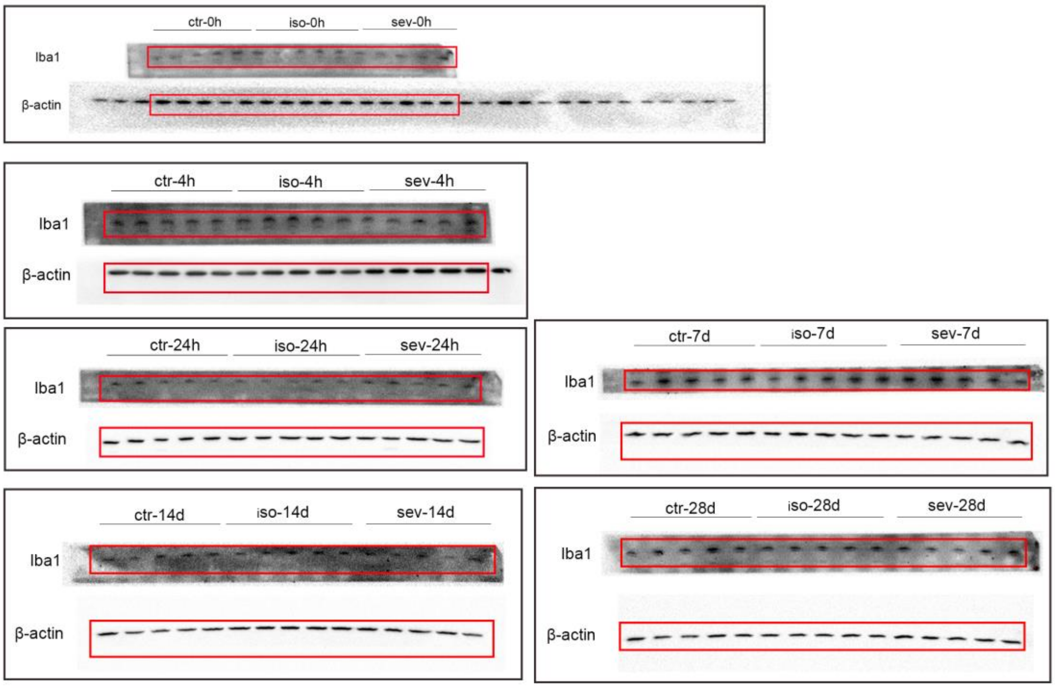

Supplementary Figure 8. Original, unprocessed blots of Supplementary Fig. 2
